# Study protocol for a multi-center, randomized controlled trial to develop Japanese denture adhesive guidelines for patients with complete dentures: the Denture Adhesive Guideline trial: study protocol for a randomized controlled trial

**DOI:** 10.1186/s13063-016-1612-x

**Published:** 2016-10-18

**Authors:** Suguru Kimoto, Yasuhiko Kawai, Atsuko Gunji, Hisatomo Kondo, Taro Nomura, Tomohiko Murakami, Akito Tsuboi, Guang Hong, Shunsuke Minakuchi, Yusuke Sato, Gaku Ohwada, Tetsuya Suzuki, Katsuhiko Kimoto, Noriyuki Hoshi, Makiko Saita, Yoshikazu Yoneyama, Yohei Sato, Masakazu Morokuma, Joji Okazaki, Takeshi Maeda, Kenichiro Nakai, Tetsuo Ichikawa, Kan Nagao, Keiko Fujimoto, Hiroshi Murata, Tadafumi Kurogi, Kazuhiro Yoshida, Masahiro Nishimura, Yasuhiro Nishi, Mamoru Murakami, Toshio Hosoi, Taizo Hamada

**Affiliations:** 1 The denture care society, Administration Office of Denture Care Society, Department of Removable Prosthodontics, Tsurumi University School of Dental Medicine, 2-1-3 Tsurumi, Tsurumi-ku, Kanagawa, 230-8501 Japan; 2 Department of Removable Prosthodontics, Nihon University School of Dentistry at Matsudo, 2-870-1 Sakaecho-nishi, Matsudo, Chiba 271-8587 Japan; 3 Department of Prosthodontics and Oral Implantology, School of Dentistry, Iwate Medical University, 19-1 Uchimaru, Morioka, 020-8505 Japan; 4 Division of Community Oral Health Science, Department of Community Medical Supports, Tohoku Medecal Megabank Organization, Tohoku University, 2-1 Seiryo-machi, Aoba-ku, Sendai, 980-8573 Japan; 5 Gerodontology and Oral Rehabilitation, Graduate School of Medical and Dental Sciences, Tokyo Medical and Dental University, 1-5-45 Yushima, Bunkyo, Tokyo, 113-8549 Japan; 6 Section of Oral Prosthetic Engineering, Tokyo Medical and Dental University, 1-5-45 Yushima, Bunkyo, Tokyo, 113-8549 Japan; 7 Division of Prosthodontics & Oral Rehabilitation, Department of Oral Function and Restoration, Graduate School of Dentistry, Kanagawa Dental University, 82 Inaoka-cho, Yokosuka, Kanagawa 238-8580 Japan; 8 Department of Removable Prosthodontics, Tsurumi University School of Dental Medicine, 2-1-3 Tsurumi, Tsurumi-ku, Kanagawa 230-8501 Japan; 9 Department of Removable Prosthodontics, Occlusion Osaka Dental University, 1-5-17 Otemae, Chuo-ku, Osaka, 540-0008 Japan; 10 Department of Oral and Maxillofacial Prosthodontics, Graduate School of Biomedical Sciences, Tokushima University, 3-18-15 Kuramoto, Tokushima, 770-8504 Japan; 11 Department of Prosthetic Dentistry, Graduate School of Biomedical Sciences, Nagasaki University, 1-7-1 Sakamoto, Nagasaki, 852-8588 Japan; 12 Department of Oral and Maxillofacial Prosthodontics, Field of Oral and Maxillofacial Rehabilitation, Course for Advanced Therapeutic, Kagoshima University Graduate School of Medical and Dental Sciences, Sakuragaoka 8-35-1, Kagoshima, 890-8544 Japan; 13 Denture Prosthodontic Restoration, Advanced Dentistry Center, Kagoshima University Medical and Dental Hospital, Sakuragaoka 8-35-1, Kagoshima, 890-8544 Japan; 14 Tsurumi University, 2-1-3 Tsurumi, Tsurumi-ku, Kanagawa 230-8501 Japan; 15 Hiroshima University, Kasumi 1-2-3, Minamiku, Hiroshima, 734-8553 Japan

**Keywords:** Complete denture, Edentulism, Denture adhesive, Retentive force, Occlusal bite force, Masticatory performance, Oral health-related quality of life

## Abstract

**Background:**

Denture adhesives, characterized as medical products in 1935 by the American Dental Association, have been considered useful adjuncts for improving denture retention and stability. However, many dentists in Japan are hesitant to acknowledge denture adhesives in daily practice because of the stereotype that dentures should be inherently stable, without the aid of adhesives. The aim of this study is to verify the efficacy of denture adhesives to establish guidelines for Japanese users. The null hypothesis is that the application of denture adhesives, including the cream and powder types, or a control (isotonic sodium chloride solution) would not produce different outcomes nor would they differentially improve the set outcomes between baseline and day 4 post-application.

**Methods:**

This ten-center, randomized controlled trial with parallel groups is ongoing. Three hundred edentulous patients with complete dentures will be allocated to three groups (cream-type adhesive, powder-type adhesive, and control groups). The participants will wear their dentures with the denture adhesive for 4 days, including during eight meals (three breakfasts, two lunches, and three dinners). The baseline measurements and final measurements for the denture adhesives will be performed on the first day and after breakfast on the fourth day. The primary outcome is a general satisfaction rating for the denture. The secondary outcomes are denture satisfaction ratings for various denture functions, occlusal bite force, resistance to dislodgement, masticatory performance, perceived chewing ability, and oral health-related quality of life. Between-subjects comparisons among the three groups and within-subjects comparisons of the pre- and post-intervention measurements will be performed. Furthermore, a multiple regression analysis will be performed. The main analyses will be based on the intention-to-treat principle. A sample size of 100 subjects per group, including an assumed dropout rate of 10 %, will be required to achieve 80 % power with a 5 % alpha level.

**Discussion:**

This randomized clinical trial will provide information about denture adhesives to complete denture wearers, prosthodontic educators, and dentists in Japan. We believe this new evidence on denture adhesive use from Japan will aid dentists in their daily practice even in other countries.

**Trial registration:**

ClinicalTrials.gov NCT01712802. Registered on 17 October 2012.

## Background

The first application of denture adhesive was reported in 1913 [1]. Hence, historically, denture adhesives have already been on the market for over 100 years. Denture adhesives, characterized as medical products in 1935 by the American Dental Association Council on Dental Materials, Instruments, and Equipment, have been considered useful adjuncts for improving denture retention and stability. However, most dentists in Japan do not acknowledge denture adhesives for several reasons. First, they believe that dentures should be stable inherently, without the aid of products such as denture adhesives. Second, famous reports have claimed that denture adhesives ruin dentures, cause shrinkage, and are harmful for denture wearers [2–5], although the quality of these studies is limited (graded as level 4 on the Oxford Levels of Evidence: does not permit clinicians to engage in evidence-based clinical decision-making) [6]. Finally, Japan has no guidelines for using denture adhesives. Consequently, Japanese dentists tend to prohibit denture wearers from using denture adhesives even though these individuals suffer from unstable dentures; moreover, many of these individuals are unable to visit a dental clinic owing to physical disabilities. Furthermore, Japanese researchers are hesitant to conduct clinical trials with denture wearers given the negative attitudes regarding denture adhesives. Thus, patients may use dental adhesives at home without understanding their effects, while dentists will not acknowledge denture adhesives without knowing their effects.

In our aging society, the number of older individuals who are unable to attend dental clinics to receive professional care is increasing. Thus, they cannot receive treatment to adjust their existing dentures or to obtain new dentures with good retention and stability, which is achieved by fitting the denture’s tissue surface to the alveolar mucosa and by sealing the peripheral border [7, 8]. Loose complete dentures can become dislodged when talking or eating, and this may induce anxiety in denture wearers, compelling them to withdraw from social activities, thus decreasing their quality of life [7]. Such patients use denture adhesives even though there are other available treatments, such as existing denture adjustment or new denture fabrication. The continuing marked increase in denture adhesive sales, which reached 12 billion yen in Japan in 2008 [9], suggests that patients use denture adhesives as an acceptable alternative to dentist-applied denture treatments. However, the use of denture adhesive remains controversial in Japan.

We reviewed the literature on denture adhesive use in patients with complete dentures when drafting the trial protocol presented herein. The effectiveness of denture adhesives has been investigated in terms of prosthesis retention, stability, masticatory function, and oral health-related quality of life (OHR-QoL). Several studies demonstrated that the retention and stability improvements were more obvious in old or ill-fitting dentures when compared to new prostheses [10–12]. However, Grasso et al. did not report any differences in adhesive-related retention and stability improvement between well-fitting and ill-fitting maxillary dentures [13]. A study by Figueiral et al., which employed vertical tensile tests and intraoral resistance transducers, showed that denture adhesives did improve maxillary complete denture retention; however, the fit of the prostheses was not reported [14]. Kapur reported that despite showing initial improvements in retention, mandibular denture wearers demonstrated significant loss of retention when asked to chew test foods and drink taste solutions [15]. Regarding bite force, several studies indicated that denture adhesives significantly improve the denture-related bite force [11, 13, 16, 17]. Using a multichannel magnetometer tracking system, Rendell et al. evaluated the effects of denture adhesives on the chewing rates in complete denture patients and found that the mean chewing rates increased immediately after the adhesive was applied and were still increased at 2 and 4 h after application [18]. Kulak et al. examined whether adhesives improve chewing ability, comfort, retention, and patient confidence using subjective measures and found positive correlations concerning the improvements after adhesive use [19]. Nicolas et al. reported that after 3 months of wearing new complete dentures, adhesives improved the subjects’ abilities to manage conventional dentures and enhanced their oral quality of life [20]. Despite the variety of studies on the use of denture adhesives, whether adhesives are beneficial to denture wearers remains controversial.

After reviewing articles selected from the National Library of Medicine’s PubMed database, Cochrane Collaboration Library, ADA Center for Evidence-Based Dentistry website, and EMBASE database, the American College of Prosthodontists published guidelines on denture adhesives [21]. The guidelines stated the following regarding the advantages of denture adhesives: (1) denture adhesives, when properly used, can improve the retention and stability of dentures and help keep food particles from accumulating beneath the dentures, even in well-fitting dentures, and (2) in a quality-of-life study, patient ratings showed that denture adhesives may improve the denture wearer’s perceptions of retention, stability, and quality of life. However, randomized controlled trials (RCTs) for denture adhesives, which can provide evidence in support of the efficacy of denture adhesives and aid in the development of denture adhesive guidelines, are rare, suggesting that even the published guidelines require further development.

While a few clinical trials have been published in Japan, they were not RCTs. Moreover, the results derived from Japanese denture wearers might be different from those mentioned in previous studies performed in other countries since patients’ attitudes toward dental materials reflect food culture and socioeconomic differences among the countries. Therefore, guidelines or official statements concerning the use of denture adhesives in clinical practice in Japan cannot be developed at this time. Performing RCTs and collecting data that can be used to develop clinical practice guidelines to standardize the usage, raise the quality of care, and reduce the risk for older Japanese individuals is essential, since there are many older individuals in Japan who cannot receive denture treatment in a dental clinic owing to their physical conditions, meaning they may turn to dental adhesives instead. Thus, we plan to perform an RCT at the following ten centers: Iwate Medical University, Tohoku University, Tokyo Medical and Dental University, Nihon University School of Dentistry at Matsudo, Tsurumi University, Kanagawa Dental College, Osaka Dental University, Tokushima University, Nagasaki University, and Kagoshima University. This ten-center RCT, which will be performed throughout Japan, has external validity. This trial is referred to as the Denture Adhesive Guideline (DAG) project. The aim of the DAG trial is to verify the efficacy of denture adhesives in order to establish guidelines for using denture adhesives in clinical practice in Japan. The null hypothesis of this study is that the application of two types of denture adhesives (cream and powder) and a control would not produce differences in the set outcomes and would not result in differences in the improvement of the set outcomes between baseline and day 4 following application.

## Methods/Design

### Organization of the Denture Adhesive Guideline trial

The Japan Denture Care Society organized the trial. The chief investigator, YK, drafted the trial protocol, which is being used at ten centers. The chief coordinators, SK and AG, maintain close contact with the coordinators at each center. Each center has a coordinator and evaluators. The coordinator controls not only the randomization (see description below) but also the schedules of the participants and evaluators. The evaluators measure the outcomes of the trials and record the data after undergoing training on how to perform the measurements. Dentists who provide the denture treatments are prohibited from serving as evaluators in the trial. The data will be sent to a third-party company for data entry. The blinded digital data will be sent back to the chief coordinator for analysis.

The DAG trial was registered in the US Clinical Trials Registry (NCT01712802) on 17 October 2012.

### Inclusion and exclusion criteria

#### Inclusion criteria

To be included in the study, the participant must be completely edentulous, willing to undergo new complete denture treatment, and willing to visit the clinic for denture adjustments as a recall patient. The protocol does not limit the inclusion of participants based on the type of chief complaint, such as pain, looseness, speech, and esthetics, among others.

#### Exclusion criteria

Participants are excluded if they: (1) are ≥90 years of age, (2) have any serious systemic illnesses that may make participating in the study difficult, (3) are unable to understand and respond to the questionnaires used in the study, (4) wear metal-based complete dentures, (5) use denture adhesives regularly, (6) have maxillofacial defects covered by prosthetics, (7) use a tissue conditioner, and/or (8) have severe oral dryness (dryness score ≤20).

### Sample size calculation

A between-group difference of 29 mm on the visual analog scale (VAS) ratings for general satisfaction was considered a meaningful difference. By using variances of 20 mm in the intervention groups and 23 mm in the control group, which were based on previously reported data [22, 23], 100 subjects per group, including an assumed dropout rate of 10 %, will be required to achieve 80 % power with an alpha level of 5 %.

### Interim analyses and early stopping of the clinical trial

The data and safety of the clinical trial will be monitored. Interim analyses of the trial will be performed when 100 and 200 participants have completed the trial. The trial will be stopped under the following conditions: (1) excess harm is observed, (2) statistically significant benefits are observed, (3) little additional useful information or negligible chance of demonstrating efficacy will be yielded if fully completed. These early stopping rules will help minimize harm and maximize the benefits for the patients enrolled in the RCT.

### Randomization, allocation concealment, and sequence generation

**Fig. 1:**
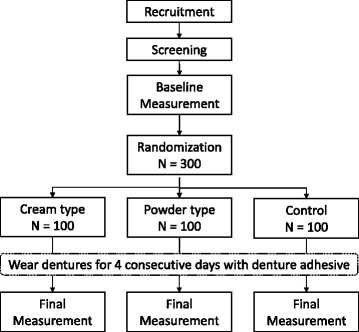
Trial flowchart

A randomized, controlled parallel clinical trial will be conducted at the abovementioned ten centers. Participants will be randomly assigned to one of the following three groups and will be instructed to apply either a denture adhesive or a control solution to both their maxillary and mandibular dentures: cream-type denture adhesive group, powder-type denture adhesive group, and control group (Fig. 1). Balanced allocation of participants to each group will be achieved using the following method: 300 random allocation numbers with no overlap and no underlap will be generated by the “RAND” and “RANK” functions in Excel (Microsoft Japan Co. Ltd., Tokyo, Japan). The first to 300^th^ numbers will be ordered randomly. Then, the first to 100^th^, 101^st^ to 200^th^, and 201^st^ to 300^th^ randomly ordered numbers will be assigned to the cream-type denture adhesive group, powder-type denture adhesive group, and control group, respectively. As per the assignment, 300 envelopes containing a card stamped with the group name will be prepared. The 300 envelopes will be divided 30 by 30 for ten centers in sequence, which means that each of the ten centers will receive 30 envelopes. Then, the coordinator at each center will open one envelope and allocate a qualifying participant to the indicated group after the patient agrees to participate in the study and provides informed consent. This method enables us to allocate the 300 numbers to the three groups equally and randomly. Once the 300 participants have been completely allocated, the number of participants in each of the three groups will be equal. Blinding the participants to the intervention is not feasible, since the participants will be able to determine the type of denture adhesive they are using during application. Evaluators are blinded to the allocation.

### Trial schedule

The trial schedule is outlined in Table 1.

**Table 1:** Schedule of participants and investigators

Trial schedule	Day 0	Day 1	Day 2	Day 3	Day 4
Visit to hospital	○				○
Instructions for denture adhesives	○				
Using denture adhesive		○	○	○	○
Eligibility screen	○				
Informed consent	○				
Patients characteristics		○			
Denture satisfaction		○			○
Oral health-related quality life		○			○
Perceived chewing ability		○			○
Perceived swallowing ability					○
Occlusal bite force		○			○
Retentive force		○			○
Masticatory performance		○			○
Oral dryness	○				○
Compliance check					○

#### Day 0

The dentists at each center will ask qualifying complete denture wearers to meet with the coordinator. The coordinator will interview the participants and explain the trial to them. Then, written informed consent will be obtained from each candidate. Once the candidate provides informed consent to the coordinator, an evaluator will perform an eligibility screening to check whether the participant meets any of the exclusion criteria. Next, the coordinator will randomly allocate the qualified candidate to one of the three trial groups using the abovementioned envelope. The participants will be asked to visit the center twice during the study.

#### Day 1

The evaluators will perform the baseline measurements listed in Table 1. The coordinator will describe how to use the denture adhesive to the participant. The participants will be instructed to apply the denture adhesive before eating breakfast, lunch, and dinner, and to remove the remaining denture adhesive immediately before applying new denture adhesive or sleeping at night. The participants will begin applying the denture adhesive before dinner on day 1, and then eat dinner with the denture adhesive applied.

#### Days 2 and 3

The participants will not visit the hospital on these days. However, they will continue to use the denture adhesive three times a day.

#### Day 4

The participants will visit the hospital after eating breakfast with the denture adhesive applied. The appointment will be scheduled so that the participants will have eaten eight meals with the adhesive applied: three breakfasts, two lunches, and three dinners. The final measurements will be performed at the hospital. The participant will give the remaining denture adhesive to the evaluator to check for compliance during the trial.

If the participants feel pain when wearing the dentures and/or have troubles with their existing dentures that require denture adjustment or repair during the trial, the dentists will adequately treat their participants in all of the allocated groups.

### Intervention

The intervention in this trial is the use of one of the following denture adhesives or control solution on the maxillary and mandibular complete dentures for four consecutive days: cream-type denture adhesive consisting of methoxyethylene/maleic anhydride copolymer, petrolatum, sodium carboxymethyl cellulose, liquid paraffin, and propyl parahydroxybenzoate (Polygrip S, GlaxoSmithKline, Tokyo, Japan); powder-type denture adhesive consisting of methoxyethylene/maleic anhydride copolymer, and sodium carboxymethyl cellulose (Polygrip powder, GlaxoSmithKline, Tokyo, Japan); or control consisting of saline solution (Isotonic Sodium Chloride Solution 20 mL CMX; Chemix Inc., Yokohama, Japan).

As mentioned above, the coordinator will instruct the participants on how to use the denture adhesive or control. The procedures for applying the powder-type denture adhesive are as follows: (1) before applying the powder, dentures should be cleaned, rinsed, and left wet; (2) gently squeeze or tap the bottle to dispense the powder onto the entire denture surface; and (3) shake off any excess powder, press the dentures into place, and hold briefly. The instructions for applying the cream-type denture adhesive are as follows: (1) before applying the cream, dentures should be cleaned, rinsed, and dried; (2) gently squeeze the tube and place the cream in small strips or a series of dots on the denture surface; and (3) press dentures firmly in place and hold briefly. Finally, the procedures for applying the control (distributed as eight bottles, with one bottle to be used per application) are as follows: (1) before applying the solution, dentures should be cleaned, rinsed, and left wet; (2) gently squeeze the bottle to dispense the liquid onto the entire denture surface; (3) press the dentures into place, and hold briefly. Compliance with the intervention with regard to using the denture adhesives and control will be checked by weighing the remaining denture adhesives and counting the remaining number of saline solution bottles, respectively.

### Primary outcome measures

Experts and members of the Japan Denture Care Society selected the outcomes for the current trial after reviewing previously published articles on denture adhesives. The primary outcome is a general satisfaction rating obtained with a 100-mm VAS. The satisfaction ratings will be determined using valid questionnaires with eight evaluation items: general satisfaction, ability to speak, ease of cleaning, stability, retention, comfort, and esthetics [24]. The participants will be asked to assess their current existing complete dentures and to record their satisfaction rating for each item on the questionnaire by drawing a vertical line on a 100-mm VAS. Each vertical line in the questionnaire represents “very satisfied” (100) at the extreme right and “very dissatisfied” (0) at the extreme left. Data will be recorded as continuous variables at 1-mm intervals using calipers, beginning at the extreme left. The general satisfaction will be measured twice: at baseline and day 4.

### Secondary outcome measures

The secondary outcomes will be measured twice: at baseline and day 4.

#### Occlusal bite force

The occlusal bite force [16, 17, 25] will be measured bilaterally at the first molar region using a force transducer occlusal force meter (GM10; Nagano Keiki, Tokyo, Japan) consisting of a digital hydraulic pressure gauge and a vinyl biting element covered with a plastic sheath. A pressure gauge displays the bite force values in Newtons on a digital screen.

#### Resistance to dislodgement

The resistance to dislodgement, which is defined as the maximum occlusal force exerted during a bite before the denture dislocates, will be measured by placing the abovementioned occlusal force meter at the incisor and canine regions. Moreover, the resistance to dislodgement quantitatively assesses the bite force needed to dislodge maxillary and/or mandibular dentures. It also demonstrates the benefits of using denture adhesive to improve the retention and stability of the tested dentures.

#### Masticatory performance

The masticatory performance, or mixing ability, will be evaluated using color-changeable chewing gum (Masticatory Performance Evaluating Gum; XYLITOL Lotte Co., Ltd., Tokyo, Japan) and a colorimeter (CR-13; Konica-Minolta Sensing, Tokyo, Japan). As per previous reports [26, 27], the measurements will be performed as follows: (1) participants will be instructed to chew the gum depending on their preferred side, without any instructions as to the chewing side, for 60 chewing cycles at a rhythm of once per second, which will be set with a metronome; (2) the chewed gum will be extracted immediately after chewing, flattened to a thickness of 1.5 mm by compressing the gum between two glass plates, and then placed on polyethylene films; and (3) the color will be measured through the polyethylene films using a colorimeter.

#### Oral health-related quality of life

The OHR-QoL will be measured with the Japanese version of the Oral Health Impact Profile for edentulous patients (OHIP-EDENT-J). The OHIP-EDENT-J is composed of 19 items distributed among the following seven subscales: functional limitations, pain, psychological discomfort, physical disability, psychological disability, social disability, and handicap [28]. Participants will be asked to rate the frequency with which they believe they encountered these items on a 5-point Likert scale, as follows: never (score 0), hardly ever (score 1), occasionally (score 2), fairly often (score 3), and very often (score 4). The sum of the ratings for all 19 items will be used as a score of OHR-QoL impairment, with the lowest score being 0 and the highest score being 76. The more the score increases, the more the OHR-QoL decreases.

#### Perceived chewing abilities

The trial will utilize two types of questionnaires to measure the patients’ self-reported chewing abilities. One of the questionnaires will measure the participants’ general perception of their chewing abilities during the mastication of each food (see list below), defined as the perceived chewing ability. The other questionnaire will measure chewing perception by asking how well the participants believe they chewed their food immediately before swallowing, defined as the perceived chewing ability at swallowing. Using the 100-mm VAS, participants will evaluate their perceived chewing ability and perceived chewing ability at swallowing when consuming the following foods: soybean curd, fish sausage, soybean sprout, cubic rice cracker, and dry squid. Each vertical line on the VAS for chewing represents “very easy to chew” (100) at the extreme right and “very difficult to chew” (0) at the extreme left, while the lines on the VAS for perceived chewing ability at swallowing represent “swallowed without chewing” at the extreme right (100) and “swallowed with chewing” (0) at the extreme left. Data will be recorded as continuous variables by measuring the distance at 1-mm intervals using calipers, beginning at the extreme left.

### Adverse events

The adverse events reported by participants are recorded on an as-needed basis.

### Covariates

The following covariates will be evaluated to determine their effects on the outcomes.

#### Participant characteristics

Participant characteristics will be evaluated using a questionnaire authorized by the Japan Prosthodontic Society [29, 30]. We will collect data on the participants’ intraoral condition-associated characteristics, i.e., on the residual ridge morphology, oral mucosa condition, maxillomandibular relationship, abnormal habits, saliva quantity and quality, and frenum attachment. In addition, data will be collected on various denture-associated characteristics, i.e., on the denture base material, artificial teeth material, denture condition, pain, retention, history of denture use, and whether the patients wear their dentures at night. Furthermore, data on the participants’ sociodemographic, medical, and anthropometric (weight, height) characteristics will be collected. The participant characteristics will be measured at baseline.

#### Oral dryness

The objective oral dryness will be measured using an oral moisture-checking device (Mucus, Life Co., Saitama, Japan). The device can measure the oral mucosal moisture level within 2 s with 200 mg of pressure. The measurements will be performed by placing a sensor vertically onto the oral mucosa until the surface of the sensor and mucosa are attached firmly. The measurement area is the lingual mucosa located 10 mm from the apex linguae [31, 32]. The oral dryness that will be used as one of the inclusion criteria will be measured at day 0 and day 4.

### Statistical analysis

First, analyses of normality using the Kolmogorov–Smirnov test will be performed for all variables. When normality can be verified, parametric tests will be performed. However, if normality cannot be verified, then two types of analyses will be performed, as follows: (1) the distributions of the dependent variables will be normalized by the appropriate transformations for parametric tests, or (2) nonparametric tests will be performed.

The between-subjects comparisons among the three groups will be performed using one-way analyses of variance with Tukey-Kramer post hoc tests or Kruskal-Wallis tests with Mann-Whitney *U* tests adjusted with the Bonferroni correction. The within-subjects comparisons of the pre- and post-intervention measurements will be performed by either paired *t* tests or Wilcoxon signed-rank tests. Furthermore, the multiple regression analysis will be performed using the abovementioned covariates as predictors.

Subgroup analyses will also be undertaken among the different subpopulations defined by each of the multiple baseline characteristics of the participants in order to study whether the effects of the intervention vary across these patient characteristics.

All of the analyses will be performed in accordance with the intention-to-treat principle, and various strategies to account for any post-randomization missing data will be contemplated. However, in the sensitivity analyses, we will also perform per protocol analyses to assess the robustness of our findings with regard to potential incomplete adherence; in particular, patients with major protocol violations or poor adherence will be excluded.

All statistical analyses will be performed using the IBM SPSS Statistics Package v21 (IBM, Armonk, NY, USA). A *p* value of <0.05 will be considered statistically significant.

## Discussion

Japan, in the midst of an aging society, has many disabled denture wearers who cannot visit a dental office even though their poor denture quality is decreasing their quality of life. Denture adhesives could help some of these patients. However, Japanese dentists hesitate to recommend or apply denture adhesives to their patients, as no Japanese guidelines for denture adhesives exist. This RCT will provide evidence of the effects of denture adhesives to complete denture wearers, caretakers at nursing homes, prosthodontic educators, and dentists in Japan. Moreover, the evidence generated by this trial in Japan will help to establish evidenced-based denture adhesive guidelines in Japan. These guidelines will release Japanese dentists from the dogma that denture adhesives do more harm than good. Furthermore, we believe the new evidence concerning the use of denture adhesives from Japan will aid dentists in their daily practice even in other countries.

### Trial status

This trial is currently ongoing. It opened to recruitment in September 2013 and aims to be completed in September 2016. Data analysis and write-up should be completed by early 2017.

## Abbreviations

DAG: Denture Adhesive Guideline
OHIP-EDENT-J: Japanese version of the Oral Health Impact Profile for edentulous patients
OHR-QoL: oral health-related quality of life
RCT: randomized controlled trial
VAS: visual analog scale
